# Optimal Concentration of Papaverine for the Inhibition of Internal Thoracic Artery Vasospasm during Coronary Artery Bypass Graft Surgery

**DOI:** 10.21470/1678-9741-2024-0058

**Published:** 2025-02-05

**Authors:** Naoko Tanaka-Totoribe, Eisaku Nakamura, Masachika Kuwabara, Shin Onizuka, Ryuichi Yamamoto

**Affiliations:** 1 First Department of Pharmacology, School of Pharmaceutical Sciences, Kyushu University of Medical Science, Nobeoka, Miyazaki, Japan; 2 Department of Cardiovascular Surgery, Miyazaki Prefectural Miyazaki Hospital, Miyazaki, Japan; 3 Kuwabara Clinic, Miyazaki, Japan; 4 School of Social Welfare, Kyushu University of Medical Science, Nobeoka, Miyazaki, Japan

**Keywords:** Coronary Artery Bypass, Thoracic Artery, Papaverine, Vasoconstriction

## Abstract

**Introduction:**

The internal thoracic artery is commonly used as a graft in coronary artery bypass grafting. In this study, we aimed to investigate whether papaverine prevents vasoconstriction caused by various vasospasm inducers, including 5-hydroxytriptamine or serotonin, in endothelium-denuded internal thoracic artery at concentrations as low as 1.25 mM used for radial arteries.

**Methods:**

Human internal thoracic artery tissue was obtained from patients (n=6) undergoing coronary artery bypass grafting. The organ bath technique was used to determine the inhibitory effects of papaverine on vasoconstriction induced by ergonovine, adenosine diphosphate, 5-hydroxytriptamine, noradrenaline, and angiotensin II in isolated endothelium-denuded internal thoracic artery. Moreover, the inhibitory effect of papaverine on collagen-stimulated human platelet aggregation was examined at the same concentration.

**Results:**

Papaverine inhibited ergonovine-induced vasoconstriction in a concentration-dependent manner. Papaverine at concentrations > 30 µM not only blocked ergonovine-induced vasoconstriction but also induced vasodilation. Papaverine at 30 µM significantly suppressed the vasoconstriction induced by 5-hydroxytriptamine or noradrenaline and completely blocked that induced by adenosine diphosphate or angiotensin II. However, 100 µM papaverine completely blocked the vasoconstriction induced by adenosine diphosphate, 5-hydroxytriptamine, noradrenaline, and angiotensin II. Additionally, papaverine significantly inhibited collagen-stimulated human platelet aggregation in a concentration-dependent manner.

**Conclusion:**

Overall, 100 µM papaverine prevented vasoconstriction by various vasospasm inducers, such as 5-hydroxytriptamine, and significantly suppressed collagen-stimulated platelet aggregation. These results suggest that papaverine at 100 µM, which is 1/10^th^ the concentration used for radial artery, is sufficient to prevent vasospasm in internal thoracic artery during coronary artery bypass grafting.

## INTRODUCTION

Coronary artery diseases, such as angina and myocardial infarction, are mainly treated via percutaneous coronary intervention, coronary artery bypass grafting (CABG), and drug treatment. In CABG, prevention of intraoperative and postoperative vascular spasms and reduction of postoperative thrombotic occlusion and stenosis prolong the patency of arteries and improve the long-term prognosis^[1]^.

The internal thoracic artery (ITA) is the most commonly used arterial graft^[2,3]^. ITA is an elastic artery with excellent long-term patency^[4,5]^. In contrast, the radial artery (RA) is less used as an arterial graft. RA has many smooth muscle cells and is prone to spasms and arteriosclerosis, warranting caution for its use. However, if used carefully, its patency rate is the same as that of ITA^[6,7]^. Prevention of vasoconstriction and spasms during and after arterial grafting is important to prevent any decrease in graft blood flow and perioperative graft patency performance.

Papaverine is widely used to prevent intraoperative vasospasm during CABG^[8,9]^. The current clinically used concentration of papaverine is 5–10 mM. However, 2 mM papaverine has been reported to impair vascular endothelial function^[10]^. Węgrzyn et al.^[10]^ reported that a low concentration (1.25 mM) of papaverine causes less damage to the vascular endothelium and is sufficient to prevent vasospasm in RA during artery grafting. When papaverine is used to prevent vasospasm, lower doses should be used to minimize the damage to vascular endothelial cells. We determined the optimal papaverine concentration for preventing ITA vasospasm. Even with caution, handling blood vessels during surgical procedures causes varying degrees of damage to vascular endothelial cells. Therefore, in this study, we used endothelium-denuded ITA to avoid any endothelial cell damage in vessels.

We hypothesized that low concentrations of papaverine, such as 1.25 mM, which are effective in RA, may prevent vasospasm in ITA.

## METHODS

### Patients

We examined the ITA segments of six patients who underwent CABG surgery. The clinical characteristics of the patients, including the body mass index, history of diabetes mellitus or myocardial infarction, and ejection fraction, are discussed in Table 1. This study was conducted according to the principles of the Declaration of Helsinki. All patients provided written informed consent to participate in the study. This study was approved by the Ethics Committees of the Miyazaki Prefectural Nobeoka Hospital and Kyushu University of Health and Welfare (approval number: 09-004).

**Table 1:** Clinical characteristics of patients enrolled in this study.

Characteristics (n = 6)	Average ± SE or n (%)
Age (years)	70.8 ± 3.0
Female sex (%)	2 (33.3)
Body mass index (Kg/m^2^)	22.1 ± 1.0
Smoking (%)	3 (50.0)
Hypertension (%)	5 (83.3)
Hyperlipidemia (%)	3 (50.0)
Diabetes mellitus (%)	2 (33.3)
Chronic renal failure (%)	0 (0.0)
Chronic obstructive pulmonary disease (%)	0 (0.0)
Cerebrovascular accident (%)	2 (33.3)
Thoracic aortic aneurysm (%)	0 (0.0)
Peripheral arterial disease (%)	2 (33.3)
Previous myocardial infarction (%)	5 (83.3)
Type of angina	
Stable (%)	6 (100.0)
Unstable (%)	0 (0.0)
Coronary lesions	
One-vessel disease (%)	0 (0.0)
Two-vessel disease (%)	2 (33.3)
Three-vessel disease (%)	4 (66.7)
Left main disease (%)	1 (16.7)
Ejection fraction (%)	54.2 ± 6.4

SE=standard error

### Preparation of Blood Vessels and Constrictive Analysis

Human ITA tissues were obtained from patients who underwent CABG at the Miyazaki Prefectural Nobeoka Hospital (Miyazaki, Japan). Portions of each ITA graft were sectioned to bypass the occluded coronary arteries, and the remaining portions were used for the experiments. The small ITA segments were immediately transported to the laboratory and placed in a modified Krebs buffer solution previously aerated with 95% O_2_ and CO_2_. The modified Krebs buffer solution (mM) consisted of 118.0 mM NaCl, 4.7 mM potassium chloride (KCl), 25.0 mM NaHCO_3_, 1.2 mM MgSO_4_, 1.1 mM KH_2_PO_4_, 2.5 mM CaCl_2_, 0.01 mM ethylenediaminetetraacetic acid, and 11.0 mM glucose (pH 7.4). After careful removal of the fat and connective tissue, the vessel was cut into 2-mm rings. The rings were denuded of the endothelium by inserting an injection needle into the lumen and gently rolling back and forth to eliminate the influence of the endothelium on the vasoconstrictive response. Constrictive responses were evaluated as previously described^[11]^. Then, cumulative concentration–response curves for ergonovine (Sigma Chemical, St. Louis, Missouri, United States of America) were plotted over a concentration range of 1–100 µM. Moreover, the inhibitory effects of papaverine (papaverine hydrochloride; 1 nM to 1 mM; Nichi-iko Co., Ltd., Toyama, Japan) on 10 µM ergonovine-induced constriction were analyzed. In another set of experiments, vehicle or papaverine (30 or 100 µM) was added to each organ bath for 30 minutes, and the rings were treated with adenosine diphosphate (ADP) (100 µM; Sigma Chemical), 5-hydroxytriptamine (5-HT) (1 µM; Sigma Chemical), noradrenaline (NA) (1 µM; Daiichi Sankyo Co., Ltd., Tokyo, Japan), or angiotensin (Ang II) (1 µM; Sigma Chemical). Isometric force measurements are given as relative values to vasoconstriction induced by 60 mM KCl added at the end of the experiment.

### Measurement of Platelet Aggregation

Blood from the veins of four healthy volunteers was collected using 21 G needles in plastic tubes containing 3.8% sodium citrate (9:1, v/v)^[12]^. The study protocol was approved by the Institutional Ethics Committee of the Miyazaki Prefectural Nobeoka Hospital and Kyushu University of Health and Welfare (approval number: 10-005). Blood samples were separated via centrifugation at 150 × g for 10 minutes to obtain the platelet-rich plasma (PRP) and at 2270 × g for 10 minutes to obtain the platelet-poor plasma (PPP). The number of platelets in PRP was adjusted to 2 × 10^5^ µL^-1^ with PPP. The adjusted PRP was further incubated with papaverine (30 or 100 µM) for five minutes at 37 °C. Then, collagen (final concentration: 1 µg/mL; Takeda Austria GmbH, A-4020 Linz, Austria) was added as an agonist. Platelet aggregation was measured using the PA-20 aggregation analyzer (Kowa, Aichi, Japan). Changes in light transmittance caused by the agonist were recorded for five minutes, and maximal aggregation was estimated. The extent of aggregation was expressed as a ratio (%) of papaverine-untreated collagen-induced platelet aggregation.

### Statistical Analyses

The number of participants in the figures indicates the number of patients studied. The data was statistically evaluated using a one-way analysis of variance followed by Dunnett’s post-hoc test. Statistical significance was set at *P*<0.05. All statistical analyses were conducted using IBM Corp. Released 2012, IBM SPSS Statistics for Windows, version 21.0, Armonk, NY: IBM Corp.

## RESULTS

**Fig. 1:**
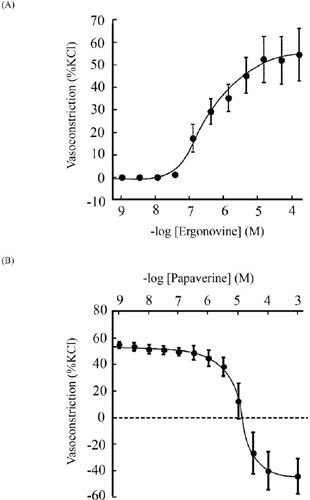
Effect of papaverine on ergonovine-induced vasoconstriction of isolated human endothelium-denuded internal thoracic artery (ITA). (a) Ergonovine (1 nM to 100 µM) induced vasoconstriction. Data are expressed as a percentage of the response to 60 mM potassium chloride (KCl) in each vessel and represented as the mean ± standard error of the mean (SEM) (n = 6). The absolute force of ITA rings induced by 60 mM KCl in the control was 1.61 ± 0.28 g (n = 6). (b) Effects of papaverine (1 nM to 1 mM) on ergonovine (10 µM)-induced vasoconstriction. Each vascular ring was preconstricted with 10 µM ergonovine, followed by the cumulative addition of papaverine (1 nM to 1 mM) to the organ bath. The vasorelaxant effect of papaverine in the presence of ergonovine was evaluated as a percentage of ergonovine (10 µM)-induced vasoconstriction before the addition of papaverine at 100% in each ring. Data are expressed as a percentage of the response to 60 mM KCl in each vessel and represented as the mean ± SEM (n = 6). The absolute force of ITA rings induced by 10 µM ergonovine was 0.75 ± 0.15 g (n = 6).

**Fig. 2:**
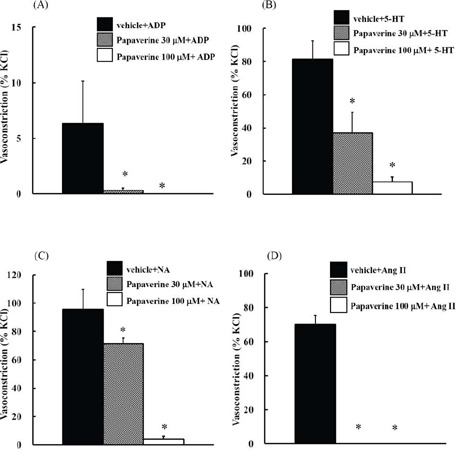
Effects of papaverine (30 and 100 µM) on adenosine diphosphate (ADP) (100 µM), 5-hydroxytriptamine (5-HT) (1 µM), noradrenaline (NA) (1 µM), and angiotensin II (Ang II) (1 µM)-induced vasoconstriction. Vehicle or papaverine (30 and 100 µM) was added to the bath for 30 minutes before the administration of ADP (100 µM) (a), 5-HT (1 µM) (b), NA (1 µM) (c), and Ang II (1 µM) (d). The ring was gradually stretched back to the optimal resting tension (2.0 g) before adding each agonist. Data are expressed as a percentage of the response to 60 mM potassium chloride (KCl) in each vessel and represented as the mean ± standard error of the mean (n = 4). The absolute force of internal thoracic artery rings induced by 60 mM KCl in the control was 1.44 ± 0.26 g (n = 4). *P<0.05 compared to each agonist.

**Fig. 3:**
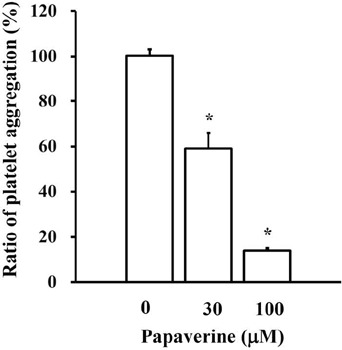
Effect of papaverine on platelet aggregation. Human platelet-rich plasma (PRP) with or without papaverine (30 and 100 µM) was stimulated with collagen (final concentration: 1 µg/mL in human PRP; n = 4). *P<0.05 compared with the control (without papaverine).

Ergonovine caused vasoconstriction in a concentration-dependent manner (1 nM to 100 µM) (Figure 1A). However, vasoconstriction induced by ergonovine (10 µM) was significantly inhibited by papaverine in a concentration-dependent manner (1 nM to 1 mM). Low concentrations of papaverine (30 µM) prevented ergonovine (10 µM)-induced vasoconstriction. High concentrations of papaverine (> 30 µM) prevented ergonovine-induced vasoconstriction and relaxed the ITA (Figure 1B). To confirm the blockade of vasoconstriction caused by various vasospasm inducers, we examined the inhibitory effects of papaverine on vasoconstriction induced by ADP, 5-HT, NA, and Ang II in isolated endothelium-denuded ITA. Figure 2 shows the inhibitory effects of papaverine on vasoconstriction induced by various vasospasm inducers. Papaverine at 30 µM significantly suppressed the vasoconstrictive response induced by 5-HT or NA and blocked that induced by ADP or Ang II. However, 100 µM papaverine completely blocked the vasoconstrictive response induced by ADP, 5-HT, NA, and Ang II (Figures2A to D). To further assess the inhibitory effect of papaverine on platelet aggregation, platelet aggregation in human PRP was measured in the presence of papaverine (30 or 100 µM) (Figure 3). Papaverine at 30 and 100 µM significantly decreased the collagen (1 µg/mL)-stimulated platelet aggregation by 60 and 14%, respectively.

## DISCUSSION

We observed that 100 µM papaverine exerted sufficient relaxing effects to prevent ITA vasospasm during CABG. This concentration is even lower (approximately 1/10) than that used in RA (1.25 mM) to suppress vasoconstriction without causing significant endothelial damage.

Ischemic heart disease is a myocardial disorder caused by myocardial ischemia and is characterized by angina pectoris and myocardial infarction. CABG is the main surgical treatment for coronary artery diseases^[13]^. Prevention of intraoperative and postoperative vascular spasms of the graft and reduction of postoperative thrombotic occlusion and stenosis prolong the graft patency and improve the long-term prognosis^[1]^. Currently, ITA and RA are commonly used as arterial grafts, and ITA is most often used as it is less likely to cause vascular spasms than RA^[14,15]^. Papaverine is used as an intraoperative antispasmodic agent and applied as a 10-fold diluted ampule stock solution (5–10 mM) to the graft^[8,9]^. However, papaverine may cause vascular endothelial cell damage and smooth muscle cell degeneration^[9,16,17]^. The concentration of the ampoule stock solution diluted 10-fold (5–10 mM) is pharmacologically extremely high, raising concerns regarding its safety. In RA, even a relatively low concentration of papaverine (1.25 mM) is sufficient to suppress vasoconstriction, with relatively little damage to vascular endothelial cells^[10]^. As ITA is less prone to vascular spasms than RA, papaverine can sufficiently suppress vascular spasms in ITA, even at a lower concentration than that used in RA. Therefore, the extent of damage to vascular endothelial cells is less in the ITA.

Ergonovine is used to induce coronary spasms during cardiac catheterization^[18]^. Moreover, it is an ergot alkaloid that stimulates the α-adrenergic and 5-HT receptors^[19]^. In this study, ergonovine induced concentration-dependent vasoconstriction in ITA, whereas papaverine dose-dependently inhibited this effect, almost completely blocking the vasoconstriction at 30 µM concentration (Figures 1A and B). ADP or 5-HT released from activated platelets stimulates the P2Y12 or 5-HT2A receptors on platelets in autocrine and paracrine manners^[20]^. After 5-HT or ADP stimulation, the aggregated platelets release large amounts of 5-HT or ADP, which subsequently induce vasoconstriction or platelet aggregation^[21,22,23]^.

NA is an endogenous catecholamine that plays a critical role in the regulation of blood pressure and metabolic functions^[24]^. Ang II is part of the renin–Ang–aldosterone system and plays an important role in regulating the vascular tone and pathogeneses of various cardiovascular diseases^[25]^. Here, we investigated the inhibitory effects of papaverine on vasoconstriction induced by various vasospasmogenic substances, mainly ADP, 5-HT, NA, and Ang II. Although papaverine at 30 µM blocked the vasoconstriction induced by ADP or Ang II, it significantly inhibited, but did not block, the vasoconstriction induced by 5-HT or NA. However, papaverine at 100 µM completely blocked the vasoconstriction induced by all the tested vasospasmogenic substances (Figures 2A to D).

A reduction in postoperative thrombotic occlusion and stenosis after CABG improves the long-term patency of bypass vessels, leading to a favorable long-term prognosis. Here, we investigated the effect of papaverine on platelet aggregation in human PRP. Collagen-stimulated human platelet aggregation was significantly inhibited by papaverine (30 and 100 µM) in a concentration-dependent manner.

Taken together, papaverine sufficiently suppressed vasospasm and platelet aggregation, even at concentrations lower than those used in RA. Moreover, papaverine suppressed the vasoconstriction and platelet aggregation induced by various vasospasmogenic substances at concentrations at least 50–100 times lower than the clinically used concentrations (5–10 mM). Therefore, the use of an appropriate papaverine concentration to prevent vasospasms ensures intraoperative safety and reduces postoperative thrombotic obstruction and stenosis. Furthermore, it suppresses platelet activation and thrombotic occlusion caused by injury to vascular endothelial cells, improves the long-term graft patency rate, and maintains the patency of bypass grafts.

## CONCLUSION

In ITA, the concentration of papaverine that almost completely suppressed vasoconstriction induced by various vasospasmogenic substances was 100 µM, which is even lower (approximately 1/10-fold) than that used to inhibit vasoconstriction in RA (1.25 mM). Therefore, 100 µM papaverine is suitable for the prevention of ITA vasospasm during CABG.

## Abbreviations, Acronyms & Symbols

ADP: Adenosine diphosphate
Ang II: Angiotensin II
CABG: Coronary artery bypass grafting
5-HT: 5-hydroxytriptamine
ITA: Internal thoracic artery
KCl: Potassium chloride
NA: Noradrenaline
PPP: Platelet-poor plasma
PRP: Platelet-rich plasma
RA: Radial artery
SE: Standard error
SEM: Standard error of the mean
